# Failed MitraClip therapy: surgical revision in high-risk patients

**DOI:** 10.1186/s13019-019-0891-1

**Published:** 2019-04-11

**Authors:** Sabreen Mkalaluh, Marcin Szczechowicz, Matthias Karck, Alexander Weymann

**Affiliations:** Department of Cardiac Surgery, Heart and Marfan Center, University of Heidelberg, Im Neuenheimer Feld 110, 69120 Heidelberg, Germany

**Keywords:** MitraClip therapy, Mitral regurgitation, Surgical therapy, Outcome, Short-term survival

## Abstract

**Background:**

MitraClip implantation is a valid interventional option that offers acceptable short-term results. Surgery after failed MitraClip procedures remains challenging in high-risk patients. The data on these cases are limited by the small sample numbers.

**Aim:**

The aim of our study is to show, that mitral valve surgery could be possible and more advantageous, even in high-risk patients.

**Methods:**

Between 2010 and 2016, nine patients underwent mitral valve surgery after failed MitraClip therapy at our institution.

**Results:**

The patients’ ages ranged from 19 to 75 years (mean: 61.2 ± 19.6 years). The median interval between the MitraClip intervention and surgical revision was 45 days (range: 0 to 1087 days). In eight of nine patients, the MitraClip intervention was initially successful and the  mitral regurgitation was reduced. Only one patient had undergone cardiac surgery previously.

Intra-operatively, leaflet perforation or rupture, MitraClip detachment, and chordal or papillary muscle rupture were potentially the causes of recurrent mitral regurgitation.

There were three early deaths. One year after surgery, the six remaining patients were alive.

**Conclusions:**

Mitral valve surgery can be successfully performed after failed MitraClip therapy in high-risk patients. The initial indication for MitraClip therapy should be considered carefully for possible surgical repair.

## Background

Mitral valve repair is the gold standard of treatment for mitral regurgitation [1, 2]. The surgery can be performed through conventional sternotomy or right anterolateral mini-thoracotomy with an excellent long-term outcome and high repair rates [3, 4]. In 2007, the percutaneous mitral valve repair via the MitraClip system was introduced in clinical use for high-risk surgical patients [5, 6]. In these high-risk patients, percutaneous mitral valve intervention showed effective reduction of mitral regurgitation [7]. Several studies reported good short-term results after mitral clipping, while others compared mitral valve surgery with MitraClip procedures. The results showed comparable outcomes, as well as significant differences in early mortality and the prevalence of serious adverse events [8–10]. In the literature, there are only few case reports and case series on surgical revision after MitraClip therapy with successful short-term results. In most cases, surgical therapy was successful despite a partially complicated and prolonged clinical course [1, 5, 11–13].

We present a report of nine patients who underwent mitral valve surgery after MitraClip therapy.

### Patients

#### Patients’ characteristics

Between 2010 and 2016, nine patients underwent mitral clipping at our hospital’s Department of Cardiology and were admitted to our Cardiac Surgery Department owing to indications of recurrent mitral regurgitation and hemodynamic instability with cardiogenic shock or cardiac arrest. Six patients had poor ejection fraction (EF) ≤ 30% and only one patient had already undergone previous cardiac surgery. All patients had histories of cardiac decompensation. At the time of surgery, six patients presented in New York Heart Association (NYHA) Class IV and the surgery was performed as ultimate ratio in these cases. Four patients suffered from chronic kidney disease without any need for dialysis. Dilated cardiomyopathy (*n* = 2) and ischemic cardiomyopathy (*n* = 3) were present in our cohort. One patient suffered from congenital mitral valve malformation and three other patients had a degenerative mitral valve regurgitation. Two patients were preoperatively ventilated. Two patients were accepted for surgery in an emergency situation.

During MitraClip intervention in one of these cases, the MitraClip catheter became locked in the mitral position. This resulted in the need for cardiopulmonary resuscitation and emergent mitral valve surgery was necessary. Another patient presented with cardiogenic shock 54 days after successful mitral clipping with mitral regurgitation (MR) reduction from IV° to III° and had to undergo emergent surgery. The preoperative conditions of the patients are summarized in Table 1.

**Table 1:** Patients Characteristics

Variable	Value (*n* = 9)
Age [mean ± SD]	61.2 ± 19.6
Sex [No.]	
Female	5 (55.6%)
Male	4 (44.4%)
NYHA class	
III	6 (66.7%)
IV	3 (33.3%)
Prior cardiac decompensation [No.]	9 (100%)
Diabetes mellitus	6 (66.7%)
Hypertension	7 (77.8%)
Smoking	5 (55.6%)
Atrial fibrillation	8 (88.9%)
Hyperlipidemia	5 (55.6%)
Prior cardiac surgery	1 (11.1%)
Time since mitral clipping [median, days]	45
Emergency surgery [No.]	2 (22.2%)
Cardiogenic shock	1 (11.1%)
Cardiac arrest with CPR	1 (11.1%)
Etiology of mitral regurgitation	
ischemic cardiomyopathy	3 (33.3%)
Congenital mitral valve malformation	1 (11.1%)
Dilated cardiomyopathy	2 (22.2%)
Degenerative, ring dilatation	2 (22.2%)
Degenerative, prolapse of anterior leaflet	1 (11.1%)
Numbers of implanted MitraClips [No.]	
1	5
2	4
Ejection fraction [No.]	
< 30%	6
30–50%	0
> 50%	3
Coronary artery disease [No.]	8 (88.9%)
Prior PTCA ± stent	4 (44.4%)
Chronic renal failure	4 (44.4%)
Dialysis	0
History of myocardial infarction [No.]	5 (55.6%)
COPD	2 (22.2%)

## Results

### Surgical data

The interval between mitral clipping and surgery ranged between zero (an emergency case with cardiac arrest) and 1087 days with eight of nine patients presenting within ≤365 days. The operations were performed using cardiopulmonary bypass through conventional sternotomy (*n* = 8) or right mini-thoracotomy (*n* = 1). The mean bypass time was 169.9 ± 36.3 min with a mean aortic clamp time of 99.6 ± 16.9 min. In only two patients, mitral valve repair was possible. They underwent mitral valve ring anuloplasty. In one case, a posterior leaflet repair in P2/P3 through triangular resection was performed and the implantation of two neochordae in segment A2/A3 was necessary. Owing to perforation, leaflet adhesion, and severe valve damage, mitral valve replacement was necessary in the seven other cases. In three cases, the closure of an iatrogenic atrial septal defect was performed. Six patients received concomitant tricuspid valve repair- four through annuloplasty and two through De Vega plasty. One patient needed coronary revascularization. An intra-aortic balloon pump (IABP) implantation was necessary in one case, and no extracorporeal membrane oxygenation (ECMO) was needed.

Intra-operatively and postoperatively there was no paravalvular leakage, and in the repair cohort the mitral regurgitation (MR) ≤ I° was noted in one case.

### Intra-operative findings

We were able to analyze the causes of failed MitraClip therapy intra-operatively. Severe valve damage caused by clipping was found in most cases. We found perforation of the posterior mitral leaflet P2 in two cases. One patient had perforation in the segment A3/P3, another had papillary muscle rupture, and in three cases the MitraClips were detached. Two patients had chordal rupture. In one of the emergent cases with perforation of the posterior mitral leaflet P2, the catheter was locked in the mitral position. No patient showed signs of endocarditis.

### Postoperative outcome

There were three in-hospital deaths (on the 6th, 17th, and 33rd days) after surgery. The patient who presented with cardiogenic shock preoperatively died on the 33rd day postoperatively due to multi-organ failure with acute renal failure, pneumonia, pancreatitis, and bowel ischemia resulting in laparotomy with bowel resection. The second patient died of hypoxemia due to aspiration of food remains on the 17th day postoperatively after an initially uneventful clinical course and the third one suffered from multiple ischemic cerebral infarctions with severe brain injury. The last one required surgery with cardiopulmonary resuscitation. Only one patient suffered from mediastinal bleeding requiring re-thoracotomy. No myocardial infarction occurred postoperatively. Respiratory failure requiring prolonged intubation (> 72 h) and tracheotomy was observed in two patients. Owing to atrioventricular block III° in three patients, pacemaker implantation was necessary. One patient received surgical revision as a consequence of severe wound infection. All six survivors were alive at the one-year follow-up.

## Discussion

Mitral valve repair remains the treatment of choice in mitral valve regurgitation [14]. Alfieri et al. demonstrated the double-orifice technique as a simple technique for repairing complex mitral valve lesions [15]. A few years later, a modified interventional technique based on the Alfieri technique, the MitraClip system, was introduced in clinical use for surgical high-risk cases [5, 16]. In the guidelines from 2012, the European Society of Cardiology and the European Association for Cardio-Thoracic Surgery recommended mitral clipping in cases of inoperability or high risk only in patients who are assessed by a heart team [17]. Comparative studies showed similar or superior results in a number of relevant endpoints of outcomes in surgical patients [10, 16, 18]. Nevertheless, until today, there is a lack of comparative studies between surgical mitral valve repair and percutaneuos mitral repair through mitral clipping with regard to long-term follow-up. Ondrus et al. compared both procedures in a small number of samples in a median follow-up time of 1028 days. The mentioned study presented similar 30-day mortality and the prevalence of serious adverse events [9]. In another comparison among octogenarians, Alozie and colleagues demonstrated better survival after 1 year and good functional results in the surgical group [18]. In conclusion, the indication for percutaneous mitral valve repair should be discussed carefully by the heart team, because the chance of successful surgical repair after failed intervention drops and the prognosis after surgery may worsen dramatically. For this reason, MitraClip intervention should not be performed in patients who can undergo surgery with acceptable surgical risk.

Furthermore, even the term “inoperable patients” needs discussing, because many cases were able to show that these inoperable patients could be operated on successfully even after failed mitral clipping [1, 12, 13, 19, 20]. A systemic review of seven studies which included 67 patients showed that the most common indication for surgical revision was recurrent mitral regurgitation greater than 2+, and in two studies one-year survival ranging from 68 to 77% was reported [21]. In our cohort, recurrent mitral regurgitation was also the most frequent indication for re-intervention. Among our study population, mitral valve repair was possible in only two cases (22%), and two of the three patients who died in the postoperative course were presented in cardiogenic shock or cardiac arrest prior to the operation. Elhmidi et al. reported a 20% (*n* = 4) mitral valve repair rate in a cohort of 25 patients undergoing surgical revision after a failed MitraClip procedure. They also showed that patients with cardiogenic shock are at the highest risk of in-hospital mortality. Interestingly, Geidel et al. showed that the number of implanted MitraClips influenced the chance of mitral repair [19]. It remains unclear in which interval surgical revision should be performed. Monsefi et al. and Geidel et al. preferred a short interval between failed interventional procedures and mitral valve (MV) surgery in order to prevent a new cardiac decompensation [5, 19].

## Conclusion

In the course of increasing popularity for interventional mitral repair, it still remains unclear how many patients with MR could be treated surgically with comparable operative risk and good functional results. The analysis of the current and here mentioned case series showed that patients who are considered as high-risk before MitraClip therapy might become suitable surgical candidates for mitral valve surgery. For that reason, we believe that an initial decision favoring a less invasive approach should be considered well, because high-risk patients thereby miss out on the prospect of valve repair.

Our present study had various limitations. The study is a retrospective analysis of a single center experience with few cases. Multicenter studies of large numbers are needed with long-term follow-up because the data on this topic are limited by case reports and small case series. We did not report the long-term outcomes.

## Abbreviations

ECMO: Extracorporeal membrane oxygenation
EF: Ejection fraction
IABP: Intra-aortic balloon pump
MR: Mitral regurgitation
MV: Mitral valve
NYHA: New York Heart Association
